# The phenotypic characteristics of polymorphonuclear neutrophils and their correlation with B cell and CD4+T cell subsets in thyroid-associated ophthalmopathy

**DOI:** 10.3389/fimmu.2024.1413849

**Published:** 2024-08-21

**Authors:** Ke Jin, Qian Yao, Bin Sun

**Affiliations:** ^1^ Department of Ophthalmology, Shanxi Eye Hospital Affiliated to Shanxi Medical University, Taiyuan, China; ^2^ Department of Ophthalmology, Shanxi Provincial People’s Hospital, Taiyuan, China

**Keywords:** thyroid-associated ophthalmopathy, immunophenotype, polymorphonuclear neutrophils, B cell, CD4+T cell

## Abstract

**Introduction:**

Thyroid-associated ophthalmopathy (TAO) is considered to be an organ-specific autoimmune disease. Polymorphonuclear neutrophils (PMN) have been implicated in the pathogenesis of TAO. However, little is known about the role of PMN in the development of TAO, much less the relationship between PMN with B cells and CD4+T cells in TAO.

**Objective:**

This study aims to investigate the phenotypic characteristics of PMN and the relationship between PMN with CD4+T cell and B cell subsets in the pathogenesis of TAO.

**Methods:**

Blood routine information was collected from 135 TAO patients, 95 Grave’s disease without TAO (GD) patients, and 116 normal controls (NC), while surface marker expression of PMN and the level of CD4+T cell and B cell subsets in peripheral blood from 40 TAO patients, 17 GD patients, and 45 NC was assessed by flow cytometry.

**Result:**

The level of PMN, CD62L+PMN, CD54+PMN, CD4+T cells, and Th17 cells displayed an increase in TAO patients than NC, while Treg cells were lower in the TAO group compared to NC. There was no statistical difference in Th1 and plasma cells among the groups. PMN were positively correlated with Th17 cells, but not the Th1, Treg, and plasma cells.

**Conclusion:**

In the present study, we found that the percentage of PMN and PMN subset cells was significantly higher in TAO than in NC, and PMN were positively correlated with Th17 cells. It suggests that PMN may be involved in the immunopathogenesis of TAO and modulate the Th17 cell response during this process.

## 1 Introduction

Thyroid-associated ophthalmopathy (TAO), also referred to as thyroid eye disease, is an organ-specific autoimmune disease that affects the orbit and ocular adnexa, leading to visual disturbances resulting from extraocular muscle involvement, proptosis, conjunctival inflammation, and edema (1). TAO is also the most frequent extrathyroidal expression of Graves’ disease and the prevalence among Graves’ patients is around 30% (2). However, owing to limited understanding of its pathogenesis, glucocorticoids remain the primary treatment for patients with active TAO, despite their modest success rate and potential serious adverse reactions (3, 4). Therefore, acquiring a comprehensive understanding of the mechanisms underlying TAO is urgently necessary to help identify appropriate treatment methods.

The main processes involved in TAO are CD4+T cell-mediated and B cell-dependent interaction. When autoimmune tolerance in TAO is disrupted, B cells process thyroid-stimulating hormone receptor peptides to activate CD4+T cells. Meanwhile, the binding of the CD40 ligand on the surface of CD4+T cell to CD40 on the B cell surface activates B cells. Activated CD4+T cells differentiate into different subsets and produce cytokines, together with autoantibodies from activated B cells, to stimulate orbital fibroblasts (OFs). These processes initiate inflammatory responses in the orbit and lead to glycosaminoglycan deposition, orbital fibrosis, and adipose hyperplasia (1, 5, 6). Although previous reports have identified important roles for CD4+ T cells, B cells, and OFs in TAO, it remains largely unknown whether other cell types are involved in TAO, and further exploration is warranted.

Polymorphonuclear neutrophils (PMN), the most abundant circulating leukocytes in humans, not only secrete factors that influence B cell maturation and plasma cell production, but also express MHCII and co-stimulatory molecules under inflammatory conditions to promote the differentiation of naïve CD4+ T cells into Th1, Th17, and regulatory T cells (Treg) in the diseases (7–12). Additionally, although no significant difference in PMN levels was found between patients with Graves' disease without thyroid-associated ophthalmopathy (GD) and normal controls (NC), PMN levels were shown to be significantly higher in patients with TAO than in both GD and NC groups and positively correlated with the clinical activity score (CAS) of TAO (13–15). Therefore, PMN may play a role in the immunopathogenesis of TAO by modulating the functions of CD4+ T cells and B cells. However, the role of PMN in TAO is not clear, and the relationship of B cells and CD4+T cells with PMN in TAO is even less well understood.

In this study, we addressed three primary lines of enquiry. First, we examined the level of PMN in patients with TAO via routine blood tests and flow cytometry. Second, we detected the expression of CD62L, CD54, CXCR2 and CXCR4 of PMN to assess neutrophil functional responses including adhesion, aggregation, phagocytosis, reactive oxygen species production, migration and senescence in TAO (16–24). Finally, we investigated the correlation of PMN with CD4+ T cell and B cell subsets. The aims of this study were to elucidate the role of neutrophils in TAO, enhance comprehension of the immunopathogenic mechanisms underlying TAO, and ultimately establish a theoretical basis for precise and early intervention strategies in future clinical practice.

## 2 Materials and methods

### 2.1 Subjects

Research subjects comprised 135 patients with TAO and 116 NC who were recruited from Shanxi Eye Hospital, and 95 patients with GD who were recruited from Shanxi Provincial People's Hospital. Data on routine blood parameters were collected from all participants. Peripheral blood samples were obtained from 40 TAO patients, 17 GD patients, and 45 NC for flow cytometry. All participants signed informed consent forms and the study protocols were approved by the Local Bioethical Committee of the Shanxi Eye Hospital and Shanxi Provincial People's Hospital.

### 2.2 The diagnosis of TAO and GD

The diagnosis of TAO was based on the European Group on Graves’ Orbitopathy (EUGOGO) consensus (25). GD was defined according to the symptoms of hyperthyroidism, and the laboratory results involve positive serum thyroid-stimulating hormone receptor antibody (TRAb, positive titer >1.75 IU/L), elevated levels of free triiodothyronine (fT3), free thyroxine (fT4), and decreased levels of thyroid-stimulating hormone (TSH). The exclusion criteria of TAO, GD, and NC groups were as follows: age <18 years, acute or chronic infection, other autoimmune diseases, or trauma history in the previous 12 months, the use of immunosuppressive therapy or radiotherapy within the previous year, participants who are pregnant, malignancy history and hematological disorders, orbital surgery, or other severe side effects to immune cells.

### 2.3 CAS

The range of activity of TAO was graded according to CAS, including seven signs, such as spontaneous retrobulbar pain, ocular pain on attempted upward or downward gaze, redness of the eyelids, redness of the conjunctiva, swelling of the eyelids, chemosis of the conjunctiva, and inflammation of caruncule and/or plica. The existence of each parameter is given a score, and the active GO is defined as CAS ≥3/7 (15, 25–27).

### 2.4 Thyroid function classification

We assessed the concentrations of TSH, fT3, and fT4 to classify individuals into three groups: patients with euthyroidism were individuals with values of TSH, fT3, and fT4 within normal ranges; hyperthyroidism involved primary (elevated fT3 and fT4 concentrations and decreased TSH level) or subclinical hyperthyroidism (decreased TSH level and fT3 and fT4 concentrations within normal ranges); and hypothyroidism included primary (elevated TSH level and decreased fT3 and fT4 concentrations) or subclinical hypothyroidism (elevated TSH level and fT3 and fT4 concentrations within normal ranges).

### 2.5 Blood routine examination

Peripheral blood samples were collected from the participants and detected using XN-9000 Sysmex (Sysmex Co., Kobe, Japan) according to the manufacturer’s instructions. The indicators obtained were as follows: white blood cell count (WBC), neutrophil count (NEUT), lymphocyte count (LYMPH), monocyte count (MONO), eosinophil count (EO), and basophil count (BASO).

### 2.6 Flow cytometry and antibodies

Peripheral blood was collected in heparin-containing tubes, and immune cells were subsequently isolated from the peripheral blood. Flow cytometric analysis was then conducted using monoclonal antibodies recognizing the antigens. Two flow cytometry panels were designed: one for granulocytes and another for lymphocytes. In the granulocyte panel, PMN (CD45+CD11b+CD66b+) was identified based on the expression of CD45, CD11b, and CD66b markers, followed by detection of PMN subsets using CD62L, CD54, CXCR2, and CXCR4 antibodies (28). In the lymphocyte panel, B cells (CD45+CD19+), plasma cells (CD45+CD19+CD138+), CD4+T cells (CD45+CD19-CD3+CD4+CD8-), Th1 cells (CD45+CD19-CD3+CD4+CD8-CXCR3+CCR6-), Th17 cells (CD45+CD19-CD3+CD4+CD8-CXCR3-CCR6+), and Treg cells (CD45+CD19-CD3+CD4+CD8-CD25+CD127low) were identified based on the expression of their markers (29–32). All antibodies were purchased from BD Biosciences (America) and the flow cytometric analyses were performed on FACS Aria III (BD Biosciences, America).

### 2.7 Statistical analysis

Statistical analyses were performed using SPSS version 25. The Student’s *T*-test was utilized to compare difference between two groups, while the one-way analysis of variance (ANOVA) was conducted on more than two groups, followed by Tukey’s *post hoc* test or least significant difference test to determine in which group the difference occurred. Chi-squared test was performed to determine sex distribution between groups. Pearson’s correlation analysis or Spearman’s correlation analysis (when the data were not normally distributed) was performed to assess the correlation between linearly related variables in cases. Continuous variables were presented as mean ± standard deviation (SD) or mean ± standard error (SEM). All *p*-values were two-tailed, and *p*-values <0.05 were considered statistically significant.

## 3 Result

### 3.1 Baseline characteristics of TAO, GD, and NC groups

The mean ages of the TAO, GD, and NC groups were 42.07 ± 14.60 years, 34.26 ± 12.04 years, and 38.97 ± 17.94 years, respectively. The mean age distribution was similar in TAO and NC groups, while the mean age of subjects in the GD group was lower than that of subjects in the TAO and NC groups. The majority of participants in the TAO, GD, and NC groups were females, and the gender distribution was similar among the three groups. The mean duration since diagnosis of Graves’ disease in the TAO and GD groups was 21.65 ± 40.58 months and 15.58 ± 30.97 months, respectively, and the mean duration since diagnosis of TAO in the TAO group was 13.67 ± 29.02 months. **Table 1** presents the general characteristics of all study participants. Among the participants in the study, 40 TAO patients, 17 GD patients and 45 NC provided peripheral blood for flow cytometry detection. **Table 2** displays general characteristics of these participants.

**Table 1:** Demographic and clinical information of all participants in the study.

Clinical parameter	TAO	GD	NC
Number of subjects	135	95	116
Age (year) (mean ± SD)	42.07 ± 14.60	34.26 ± 12.04	38.97 ± 17.94
Sex (F/M)	83/52	71/24	71/45
Months since the diagnosis of Graves’ disease (mean ± SD)	21.65 ± 40.58	15.58 ± 30.97	–
Months since the diagnosis of TAO (mean ± SD)	13.67 ± 29.02	–	–
Current thyroid state			
Euthyroid	49	9	116
Hyperthyroid	64	85	0
Hypothyroid	22	1	0
Smoking history	53	21	30
CAS ≥3	23	–	–

**Table 2:** Demographic and clinical information of the participants who provided peripheral blood for flow cytometry detection.

Clinical parameter	TAO	GD	NC
Number of subjects	40	17	45
Age (year) (mean ± SD)	44.33 ± 12.73	35.18 ± 13.77	50.84 ± 18.36
Sex (F/M)	13/27	15/2	25/20
Months since the diagnosis of Graves’ disease (mean ± SD)	18.12 ± 22.64	16.65 ± 27.88	–
Months since the diagnosis of TAO (mean ± SD)	12.61 ± 20.50	–	–
Current thyroid state			
Euthyroid	14	2	45
Hyperthyroid	16	15	0
Hypothyroid	10	0	0
Smoking history	22	2	13
CAS ≥3	6	–	–

### 3.2 Differences in PMN according to blood routine results

**Figure 1:**
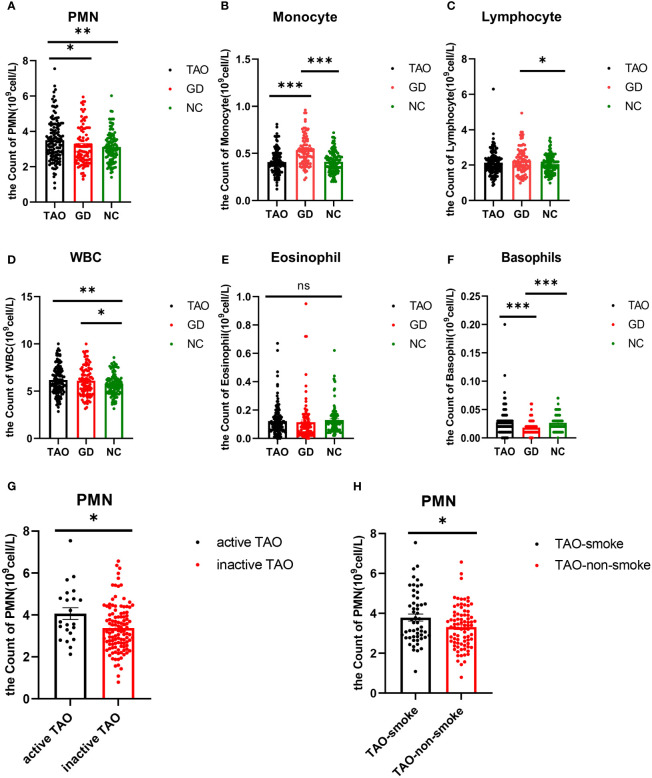
Analysis of PMN, monocyte, lymphocyte, WBC, eosinophil, and basophil counts in TAO, GD, and NC groups by blood routine information. Statistical analysis of PMN, monocyte, lymphocyte, WBC, eosinophil, and basophil counts in the three groups is shown **(A–F)**. Statistical analysis of PMN cell count in active TAO and inactive TAO was shown **(G)**. Statistical analysis of PMN cell count in TAO with and without smoking history is shown **(H)**. Data are represented as mean ± SEM. *P < 0.05, **P < 0.01, ***P < 0.001, ns, No significance.

Regarding hematological parameters, the count of PMN was significantly higher in the TAO group compared to the GD group and NC group (**Table 3** and **Figure 1A**). Patients with active TAO had higher PMN counts than inactive TAO patients, and a higher PMN count was observed in TAO patients with smoking history than in TAO patients without smoking history (**Figures 1G, H**). Besides this, we also found that the count of WBC was significantly higher in the TAO and GD groups (**Figure 1D** and **Table 3**) than that in NC, while there were notable elevations or reductions in monocyte, lymphocyte, and basophil levels within the GD group compared with the NC group (**Figures 1B, C, F**; **Table 3**). However, no significant difference was found in eosinophil levels among the TAO, GD, and NC groups (**Figure 1E**).

**Table 3:** Baseline characteristics of blood routine data among the three groups.

Variables	Group 1 TAO	Group 2 GD	Group 3 NC	*p* (groups 1 and 2)	*p* (groups 1 and 3)	*p* (groups 2 and 3)
WBC (10^9^/L)	6.18 ± 1.53	6.10 ± 1.58	5.73 ± 1.14	NS	<0.01	<0.05
NEUT (10^9^/L)	3.50 ± 1.18	3.19 ± 1.10	3.11 ± 0.85	<0.05	<0.01	NS
LYMPH (10^9^/L)	2.13 ± 0.67	2.25 ± 0.76	2.05 ± 0.52	NS	NS	<0.05
EO (10^9^/L)	0.12 ± 0.11	0.11 ± 0.15	0.12 ± 0.10	NS	NS	NS
BASO (10^9^/L)	0.03 ± 0.02	0.02 ± 0.01	0.03 ± 0.01	<0.001	NS	<0.001
MONO (10^9^/L)	0.41 ± 0.13	0.54 ± 0.16	0.41 ± 0.11	<0.001	NS	<0.001

TAO, n = 135; GD, n = 95; NC, n = 116. Data are presented as mean ± SD. The one-way analysis of variance (ANOVA) was conducted on more than two groups, followed by Tukey’s post hoc test or least significant difference test to determine in which group the difference occurred. P-value <0.05 was considered to be significant.

WBC, white blood cell count; NEUT, neutrophil count; LYMPH, lymphocyte count; EO, eosinophil count; BASO, basophil count; MONO, monocyte count; NS, no significance.

### 3.3 Differences in PMN and their subsets among TAO, GD, and NC groups according to flow cytometry

**Figure 2:**
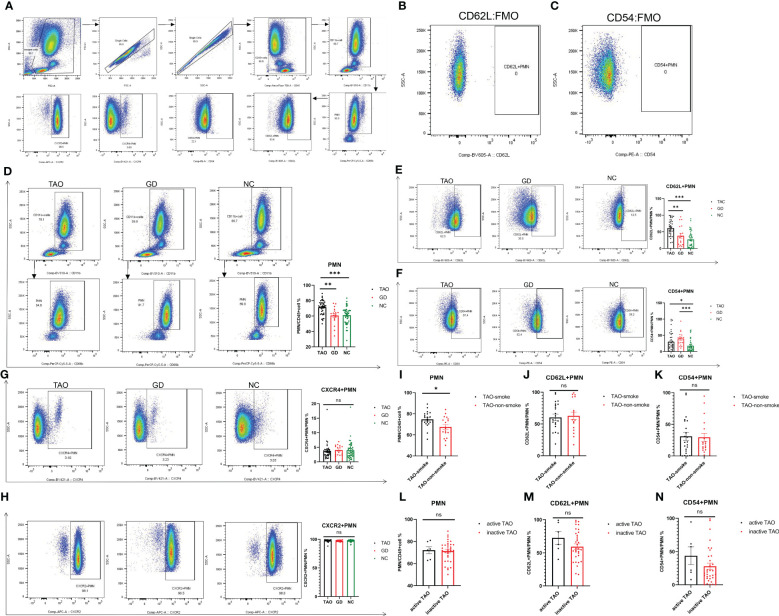
Flow cytometry analysis of PMN, CD62L+PMN, CD54+PMN, CXCR2+PMN, and CXCR4+PMN cells in participants with TAO, GD, and NC. Gating of PMN, CD62L+PMN, CD54+PMN, CXCR2+PMN, and CXCR4+PMN cells was represented using flow cytometry for each group **(A)**. Gating of CD62L+PMN and CD54+PMN by using Fluorescence Minus One controls **(B, C)**. Statistical analysis of PMN, CD62L+PMN, CD54+PMN, CXCR2+PMN, and CXCR4+PMN percentages in the three groups is shown **(D–H)**. Statistical analysis of PMN, CD62L+PMN, and CD54+PMN cell percentages in TAO with and without smoking history is shown **(I–K)**. Statistical analysis of PMN, CD62L+PMN, and CD54+PMN cell percentages in active TAO and inactive TAO is shown **(L–N)**. Data are represented as mean ± SEM. *P < 0.05, **P < 0.01, ***P < 0.001, ns, No significance.

Multi-parameter flow cytometry was used to examine the proportion of PMN and their surface markers (CD62L, CD54, CXCR2, and CXCR4) in the TAO, GD, and NC groups. PMN were identified by the expression of CD11b and CD66b (**Figure 2A**). CD62L+PMN and CD54+PMN were plotted and analyzed by using Fluorescence Minus One controls (**Figures 2B, C**). Consistent with the change trend in blood routine examination, the proportion of PMN in the TAO group was significantly higher than that in the NC and GD groups (**Figures 2D**), and the proportion of PMN in the TAO group with smoking history was higher than that in the TAO group without smoking history (**Figure 2I**), but PMN did not differ between the active TAO group and the inactive TAO group, which may be due to the fact that the active TAO patients in our study were too few (**Figure 2L**). In addition, the percentage of PMN cells expressing CD62L and CD54 varied among groups. The fraction of PMN cells from patients with TAO expressing CD62L was significantly greater than that of NC and GD PMN cells (**Figure 2E**), while the fraction of PMN cells from patients with TAO and GD expressing CD54 increased significantly compared with NC (**Figure 2F**). However, there was no significant difference in the level of CD54 and CD62L between active TAO and inactive TAO (**Figures 2M, N**), as well as between TAO with and without smoking history (**Figures 2J, K**). In additon, we also observed that there was no significant difference in the level of CXCR2 and CXCR4 among the groups (**Figures 2G, H**).

### 3.4 Differences in B cell and CD4+T cell subsets among the TAO, GD, and NC groups according to flow cytometry

**Figure 3:**
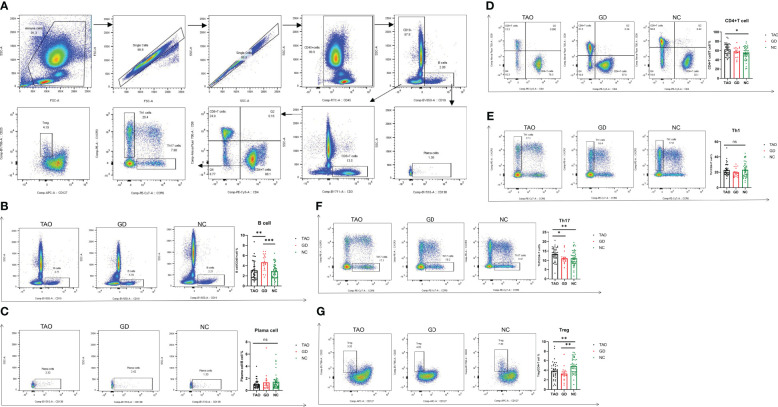
Flow cytometry analysis of Th1, Th17, Treg, CD4+T cell, B cell, and plasma cell percentages in patients with TAO, GD, and NC. Gating of Th1, Th17, Treg, CD4+T cells, B cells, and plasma cells by flow cytometry is presented **(A)**. Statistical analysis of Th1, Th17, Treg, CD4+T cell, B cell, and plasma cell percentages in the three groups is shown **(B–G)**. Data are represented as mean ± SEM. *P < 0.05, **P < 0.01, ***P < 0.001, ns, No significance.

To evaluate the levels of B cell and CD4+T cell subsets in TAO patients, the proportion of CD4+T cells, Th1, Th17, Treg, B cells, and plasma cells, respectively, was detected by flow cytometry in the TAO, GD, and NC groups, and the flow gate strategy is shown in **Figure 3A**. The proportion of CD4+T cells and Th17 cells in the TAO group was significantly higher than that in the NC group (**Figures 3D, F**), while the proportion of Treg cells in the TAO and GD groups decreased significantly compared to the NC (**Figure 3G**). In addition, we also found that the proportion of B cells was significantly increased in the GD group compared with the TAO and NC groups (**Figure 3B**). However, there was no significant difference in the proportion of Th1 cells and plasma cells among the TAO, GD, and NC groups (**Figures 3C, E**).

### 3.5 The correlation of PMN with B cell and T cell subsets among the TAO, GD, and NC groups

**Figure 4:**
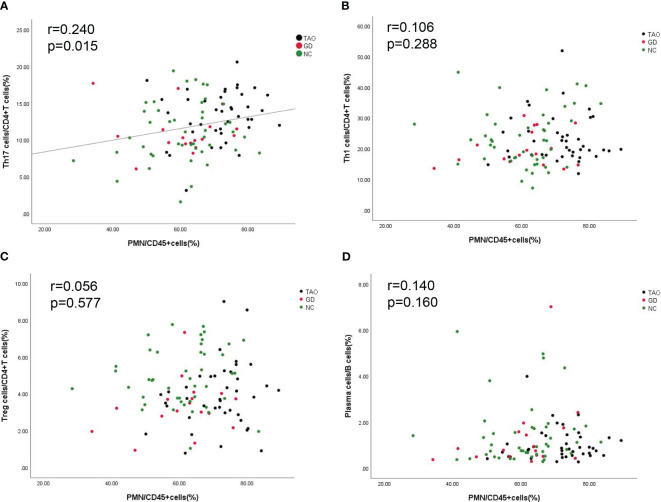
Correlation between PMN with Th1 cells, Th17 cells, Treg cells, and plasma cells. The correlation between PMN with Th1 cells, Th17 cells, Treg cells, and plasma cells is shown **(A–D)**. *P*-value <0.05 was considered to be significant.

In order to examine the potential link between PMN with immune activation, we assessed the association between PMN with B cell and CD4+T cell subsets. The result showed that PMN were positively correlated with Th17 cells (*r* = 0.240, *p* < 0.05) (**Figure 4A**) but not with Th1, Treg, and plasma cells (**Figures 4B-D**).

## 4 Discussion

The stability and balance of PMN cells are the important factors for the body’s immune function (33). In the present study, we assessed PMN and the B cell and CD4+T cell subsets in TAO patients. We found that the level of PMN and the surface expression of CD62L and CD54 were significantly higher in TAO patients than that in NC. We also found that PMN were positively correlated with Th17 cells. It suggests that PMN may be involved in the pathogenesis of TAO and modulate the response of Th17 cells in the process of TAO. This is the first study to explore the relationship of PMN with CD4+T cell and B cell subsets in TAO.

PMN account for 50–70% of all leukocytes in peripheral blood and play an important role in the activation and regulation of innate and adaptive immunity (7, 34, 35). In 2017, T Celik found that in patients with TAO, the neutrophil count was elevated compared with that in NC (14). Subsequently, Joanna Szydełko el also found that neutrophils were elevated in TAO patients and positively correlated with the CAS of these patients (15). Our findings are in agreement with these reports. Besides, higher levels of CD62L and CD54 were found to be expressed by PMN from patients with TAO compared with PMN from NC. This is the first study to explore the expression of CD62L and CD54 in PMN from patients with TAO. L-selectin (CD62L) is a constitutively expressed adhesion molecule on the surface of neutrophils that mediates low-affinity binding and rolling along the endothelium. L-selectin shedding occurs as neutrophils transmigrate into tissues (16, 17). Given this finding, our study showed that patients with TAO had higher L-selectin expression on PMN, which suggested that they had a higher capacity for margination. Intercellular adhesion molecule-1 (ICAM-1) (CD54) is a cell-surface glycoprotein implicated in leukocyte trafficking, immune cell effector functions, clearance of pathogens and dead cells, and T-cell activation (18). ICAM-1 expression increased on PMN from patients with TAO, which suggested that PMN from these patients not only exhibited enhanced reactive oxygen species generation, improved phagocytosis, and elevated levels of endotoxin, but also bound to the lymphocyte function associated antigen-1 of T cells to affect T-cell activation (18–21). Therefore, we speculate that PMN plays an important role in the pathogenesis of TAO.

CD4+T cell and B cell subsets are the main effector cells in TAO. In our study, consistent with a previous report, TAO patients exhibited a significant increase in CD4+ T cells and Th17 cells compared with the NC group, while the percentage of Treg cells was lower in the TAO and GD groups than NC (36–38). No significant difference was found in Th1 cells among the TAO, GD, and NC groups, which was consistent with the fact that Th1 was predominantly involved in active TAO, but most of the TAO patients in our study were inactive TAO (5, 39, 40). In addition, B cells and plasma cells did not differ between the TAO and NC groups. Based on this finding, we speculate that although orbital infiltrating B cells seem to play a pathogenetic role in TAO, peripheral B cells may have a marginal role with the presence and the clinical features of TAO.

Previous reports had found that neutrophils could activate and regulate Th17 cell-driven adaptive immune responses by expressing a wide range of cytokines and immunosuppressive and immunostimulatory molecules (8, 41). Our study also found that PMN were positively correlated with Th17 cells. Therefore, we speculate that PMN may modulate Th17 cells in the pathogenesis of TAO, whereas the mechanism of PMN on Th17 cells in TAO remains largely unknown, which needs further investigation. PMN were not correlated with Th1 cells, and this might be due to the level of Th1 cells, which was not elevated in the TAO group compared to the GD and NC groups.

There are still some limitations in our study. First, the sample in the study is small, so we need a large and more patient population, especially active TAO patients, to replicate our results. Second, our research is only a clinical study, and does not explore the functions of PMN in TAO or the underlying mechanisms. Future animal or human experiments are required to explore the potential mechanism underlying the role of PMN in TAO. Third, because many male patients with TAO donated blood draws and participated in flow cytometry testing, there were more males in the TAO flow cytometry group than in the other groups. In future, more female TAO patients should be included in flow cytometry testing. Fourth, the mean age of patients in the enrolled GD group was lower than in the TAO and NC groups, which may influence the findings of the study. A larger number of GD patients, particularly those belonging to older age groups, is necessary to enhance the reproducibility of the findings. Finally, our study does not involve Th2 cells, and future experiments are needed to investigate the relationship between PMN and Th2 cells in TAO.

## Data Availability

The datasets presented in this study can be found in online repositories. The names of the repository/repositories and accession number(s) can be found in the article/**Supplementary Material**.

## References

[1] Lee ACH Kahaly GJ. Pathophysiology of thyroid-associated orbitopathy. Best Pract Res Clin Endocrinol Metab. (2023) 37(2):101620. doi: 10.1016/j.beem.2022.101620 35181241

[2] Bartalena L Tanda ML. Current concepts regarding graves' orbitopathy. J Intern Med. (2022) 292(5):692–716. doi: 10.1111/joim.13524 35604323 PMC9796560

[3] Gonzalez-Garcia A Sales-Sanz M. Treatment of graves' ophthalmopathy. Med Clin (Barc). (2021) 156(4):180–6. doi: 10.1016/j.medcli.2020.07.031 33069387

[4] Smith TJ Kahaly GJ Ezra DG Fleming JC Dailey RA Tang RA. Teprotumumab for thyroid-associated ophthalmopathy. N Engl J Med. (2017) 376(18):1748–61. doi: 10.1056/NEJMoa1614949 PMC5718164 28467880

[5] Fang S Lu Y Huang Y Zhou H Fan X. Mechanisms that underly t cell immunity in graves' orbitopathy. Front Endocrinol (Lausanne). (2021) 12:648732. doi: 10.3389/fendo.2021.648732 33868176 PMC8049604

[6] Salvi M Covelli D. B cells in graves' orbitopathy: more than just a source of antibodies? Eye (Lond). (2019) 33(2):230–4. doi: 10.1038/s41433-018-0285-y PMC6367428 30514895

[7] Herrero-Cervera A Soehnlein O Kenne E. Neutrophils in chronic inflammatory diseases. Cell Mol Immunol. (2022) 19(2):177–91. doi: 10.1038/s41423-021-00832-3 PMC8803838 35039631

[8] Kvedaraite E. Neutrophil-t cell crosstalk in inflammatory bowel disease. Immunology. (2021) 164(4):657–64. doi: 10.1111/imm.13391 PMC8561100 34240423

[9] Costa S Bevilacqua D Cassatella MA Scapini P. Recent advances on the crosstalk between neutrophils and b or t lymphocytes. Immunology. (2019) 156(1):23–32. doi: 10.1111/imm.13005 30259972 PMC6283649

[10] Fanger NA Liu C Guyre PM Wardwell K O'Neil J Guo TL. Activation of human t cells by major histocompatability complex class II expressing neutrophils: proliferation in the presence of superantigen, but not tetanus toxoid. Blood. (1997) 89(11):4128–35. doi: 10.1182/blood.V89.11.4128 9166855

[11] Radsak M Iking-Konert C Stegmaier S Andrassy K Hansch GM. Polymorphonuclear neutrophils as accessory cells for t-cell activation: major histocompatibility complex class II restricted antigen-dependent induction of t-cell proliferation. Immunology. (2000) 101(4):521–30. doi: 10.1046/j.1365-2567.2000.00140.x PMC2327116 11122456

[12] Scapini P Bazzoni F Cassatella MA. Regulation of b-cell-activating factor (BAFF)/B lymphocyte stimulator (BLyS) expression in human neutrophils. Immunol Lett. (2008) 116(1):1–6. doi: 10.1016/j.imlet.2007.11.009 18155301

[13] Turan E. Evaluation of neutrophil-to-lymphocyte ratio and hematologic parameters in patients with graves' disease. Bratisl Lek Listy. (2019) 120(6):476–80. doi: 10.4149/BLL_2019_076 31223030

[14] Celik T. Neutrophil-to-lymphocyte ratio in thyroid ophthalmopathy. Bratisl Lek Listy. (2017) 118(8):495–8. doi: 10.4149/BLL_2017_095 29050489

[15] Szydelko J Litwinczuk M Szydelko M Matyjaszek-Matuszek B. Neutrophil-to-Lymphocyte, monocyte-to-Lymphocyte and platelet-to-Lymphocyte ratios in relation to clinical parameters and smoking status in patients with graves' orbitopathy-novel insight into old tests. J Clin Med. (2020) 9(10). doi: 10.3390/jcm9103111 PMC7600876 32993174

[16] Ivetic A. A head-to-tail view of l-selectin and its impact on neutrophil behaviour. Cell Tissue Res. (2018) 371(3):437–53. doi: 10.1007/s00441-017-2774-x PMC5820395 29353325

[17] Ivetic A Hoskins Green HL Hart SJ. L-selectin: A major regulator of leukocyte adhesion, migration and signaling. Front Immunol. (2019) 10:1068. doi: 10.3389/fimmu.2019.01068 31139190 PMC6527602

[18] Bui TM Wiesolek HL Sumagin R. ICAM-1: A master regulator of cellular responses in inflammation, injury resolution, and tumorigenesis. J Leukoc Biol. (2020) 108(3):787–99. doi: 10.1002/JLB.2MR0220-549R PMC7977775 32182390

[19] Woodfin A Beyrau M Voisin MB Ma B Whiteford JR Hordijk PL. ICAM-1-expressing neutrophils exhibit enhanced effector functions in murine models of endotoxemia. Blood. (2016) 127(7):898–907. doi: 10.1182/blood-2015-08-664995 26647392 PMC4863345

[20] Wang JH Sexton DM Redmond HP Watson RW Croke DT Bouchier-Hayes D. Intercellular adhesion molecule-1 (ICAM-1) is expressed on human neutrophils and is essential for neutrophil adherence and aggregation. Shock. (1997) 8(5):357–61. doi: 10.1097/00024382-199711000-00007 9361346

[21] Takashi S Okubo Y Horie S. Contribution of CD54 to human eosinophil and neutrophil superoxide production. J Appl Physiol (1985). (2001) 91(2):613–22. doi: 10.1152/jappl.2001.91.2.613 11457772

[22] Hu N Westra J Rutgers A Doornbos-Van der Meer B Huitema MG Stegeman CA. Decreased CXCR1 and CXCR2 expression on neutrophils in anti-neutrophil cytoplasmic autoantibody-associated vasculitides potentially increases neutrophil adhesion and impairs migration. Arthritis Res Ther. (2011) 13(6):R201. doi: 10.1186/ar3534 22152684 PMC3334654

[23] Sawant KV Xu R Cox R Hawkins H Sbrana E Kolli D. Chemokine CXCL1-mediated neutrophil trafficking in the lung: Role of CXCR2 activation. J Innate Immun. (2015) 7(6):647–58. doi: 10.1159/000430914 PMC4618178 26138727

[24] De Filippo K Rankin SM. CXCR4, the master regulator of neutrophil trafficking in homeostasis and disease. Eur J Clin Invest.. (2018) 48 Suppl 2(Suppl Suppl 2):e12949. doi: 10.1111/eci.12949 29734477 PMC6767022

[25] Bartalena L Baldeschi L Boboridis K Eckstein A Kahaly GJ Marcocci C. The 2016 european thyroid Association/European group on graves' orbitopathy guidelines for the management of graves' orbitopathy. Eur Thyroid J. (2016) 5(1):9–26. doi: 10.1159/000443828 27099835 PMC4836120

[26] Barrio-Barrio J Sabater AL Bonet-Farriol E Velazquez-Villoria A Galofre JC. Graves' ophthalmopathy: VISA versus EUGOGO classification, assessment, and management. J Ophthalmol. (2015) 2015:249125. doi: 10.1155/2015/249125 26351570 PMC4553342

[27] Bartalena L Kahaly GJ Baldeschi L Dayan CM Eckstein A Marcocci C. The 2021 european group on graves' orbitopathy (EUGOGO) clinical practice guidelines for the medical management of graves' orbitopathy. Eur J Endocrinol. (2021) 185(4):G43–67. doi: 10.1530/EJE-21-0479 34297684

[28] Lakschevitz FS Hassanpour S Rubin A Fine N Sun C Glogauer M. Identification of neutrophil surface marker changes in health and inflammation using high-throughput screening flow cytometry. Exp Cell Res. (2016) 342(2):200–9. doi: 10.1016/j.yexcr.2016.03.007 26970376

[29] Wang SR Zhong N Zhang XM Zhao ZB Balderas R Li L. OMIP 071: A 31-parameter flow cytometry panel for in-depth immunophenotyping of human t-cell subsets using surface markers. Cytometry A. (2021) 99(3):273–7. doi: 10.1002/cyto.a.24272 33219622

[30] Anderson J Jalali S Licciardi PV Pellicci DG. OMIP-91: A 27-color flow cytometry panel to evaluate the phenotype and function of human conventional and unconventional t-cells. Cytometry A. (2023) 103(7):543–7. doi: 10.1002/cyto.a.24738 37183268

[31] Bonecchi R Bianchi G Bordignon PP D'Ambrosio D Lang R Borsatti A. Differential expression of chemokine receptors and chemotactic responsiveness of type 1 t helper cells (Th1s) and Th2s. J Exp Med. (1998) 187(1):129–34. doi: 10.1084/jem.187.1.129 PMC2199181 9419219

[32] Cossarizza A Chang HD Radbruch A Abrignani S. Guidelines for the use of flow cytometry and cell sorting in immunological studies (third edition). Eur J Immunol. (2021) 51(12):2708–3145. doi: 10.1002/eji.202170126 PMC11115438 34910301

[33] Bert S Nadkarni S Perretti M. Neutrophil-t cell crosstalk and the control of the host inflammatory response. Immunol Rev. (2023) 314(1):36–49. doi: 10.1111/imr.13162 36326214 PMC10952212

[34] Soehnlein O Steffens S Hidalgo A Weber C. Neutrophils as protagonists and targets in chronic inflammation. Nat Rev Immunol. (2017) 17(4):248–61. doi: 10.1038/nri.2017.10 28287106

[35] Mantovani A Cassatella MA Costantini C Jaillon S. Neutrophils in the activation and regulation of innate and adaptive immunity. Nat Rev Immunol. (2011) 11(8):519–31. doi: 10.1038/nri3024 21785456

[36] Vitales-Noyola M Ramos-Levi AM Martinez-Hernandez R Serrano-Somavilla A Sampedro-Nunez M Gonzalez-Amaro R. Pathogenic Th17 and Th22 cells are increased in patients with autoimmune thyroid disorders. Endocrine. (2017) 57(3):409–17. doi: 10.1007/s12020-017-1361-y 28669056

[37] Zhao J Lin B Deng H Zhi X Li Y Liu Y. Decreased expression of TIM-3 on Th17 cells associated with ophthalmopathy in patients with graves' disease. Curr Mol Med. (2018) 18(2):83–90. doi: 10.2174/1566524018666180705105753 29974826 PMC6128070

[38] Li C Yuan J Zhu YF Yang XJ Wang Q Xu J. Imbalance of Th17/Treg in different subtypes of autoimmune thyroid diseases. Cell Physiol Biochem. (2016) 40(1-2):245–52. doi: 10.1159/000452541 27855396

[39] Aniszewski JP Valyasevi RW Bahn RS. Relationship between disease duration and predominant orbital t cell subset in graves' ophthalmopathy. J Clin Endocrinol Metab. (2000) 85(2):776–80. doi: 10.1210/jcem.85.2.6333 10690890

[40] Park M Banga JP Kim GJ Kim M Lew H. Human placenta-derived mesenchymal stem cells ameliorate orbital adipogenesis in female mice models of graves' ophthalmopathy. Stem Cell Res Ther. (2019) 10(1):246. doi: 10.1186/s13287-019-1348-0 31399042 PMC6688254

[41] Fan X Shu P Wang Y Ji N Zhang D. Interactions between neutrophils and t-helper 17 cells. Front Immunol. (2023) 14:1279837. doi: 10.3389/fimmu.2023.1279837 37920459 PMC10619153

